# Tobacco control policies that influence Filipino adults who smoke: Results from a discrete choice experiment

**DOI:** 10.18332/tid/217005

**Published:** 2026-03-10

**Authors:** Lauren Czaplicki, Elizabeth Crespi, Raniyan Zaman, Farahnaz Islam, Joanna E. Cohen, Ana Maria Felisa G. Mayor, Filomena T. Dayagbil, Kevin Welding

**Affiliations:** 1 Johns Hopkins Bloomberg School of Public Health Institute for Global Tobacco Control, Baltimore, United States; 2 Tempus AI, Chicago, United States; 3 GoodThinking Inc., City of Pasig, The Philippines; 4 College of Teacher Education, Cebu Normal University, Cebu City, The Philippines

**Keywords:** health warning labels, taxation, plain packaging, discrete choice experiment, Philippines

## Abstract

**INTRODUCTION:**

In the Philippines, approximately 13 million adults smoke. Policies to raise cigarette taxes, increase health warning label (HWL) size, and restrict menthol could reduce smoking.

**METHODS:**

We conducted an online discrete choice experiment (DCE) in November 2023 to assess hypothetical policy responses among 886 Filipino adults who smoke across three regions of the Philippines (Luzon, Visayas, Mindanao). The DCE included ten choice sets of three cigarette packs. Packs varied on excise tax [60 (ref.), 70, 80, 90 pesos (PHP)], packaging [domestic packs: 50% HWL branded (ref.), 50% HWL plain, 85% HWL branded, 85% HWL plain; foreign pack, no HWL], and flavor [tobacco (ref.), menthol]. Participants were randomly assigned to see one choice set using a programmed 1:1 randomization ratio and asked to select which pack: 1) they would choose, 2) would make them most consider quitting, and 3) would be less harmful.

**RESULTS:**

Participants had lower odds of choosing packs with a 70 (adjusted odds ratio, AOR=0.58; 95% CI: 0.45–0.74), 80 (AOR=0.33; 95% CI: 0.25–0.43), or 90 (AOR=0.24; 95% CI: 0.18–0.33) PHP excise tax, and higher odds (AOR=4.02; 95% CI: 2.86–5.66) of choosing the foreign branded pack with no HWL. For quitting, participants had higher odds of selecting packs with an 80 (AOR=1.53; 95% CI: 1.18–1.99) or 90 (AOR=1.94; 95% CI: 1.50–2.51) PHP tax and 3.40 (95% CI: 2.58–4.46) and 3.30 (95% CI: 2.42–4.49) higher odds of selecting a branded or plain pack with 85% HWL coverage, respectively. Participants had lower odds of thinking packs with a 70 (AOR=0.69; 95% CI: 0.52–0.92), 80 (AOR=0.73; 95% CI: 0.56–0.96), or 90 (AOR=0.67; 95% CI: 0.50–0.89) PHP excise tax were less harmful and higher odds (AOR=5.21; 95% CI: 3.70–7.32) of thinking the foreign pack was less harmful.

**CONCLUSIONS:**

Results suggest raising the excise tax to at least 80 PHP and implementing 85% HWLs may encourage smoking cessation in the Philippines. Our findings can be used by policymakers and advocates in the Philippines to implement strong tobacco control policies to protect public health.

## INTRODUCTION

Approximately 13 million adults in the Philippines smoke manufactured cigarettes^1^. Although rates of smoking have decreased over time from 28.3% in 2009 to 18.5% in 2021^1,2^, smoking prevalence in the Philippines remains one of the highest in the Western Pacific region^3^. In 2005, the Philippines ratified the World Health Organization’s Framework Convention on Tobacco Control (WHO FCTC)^4^, a global public health treaty that outlines evidence-based practices to reduce the supply and demand of tobacco^5^. The decline in smoking in the Philippines can be attributed, in part, to the implementation of tobacco control laws, many of which are based on FCTC recommendations^6-8^. These include raising the price of cigarettes through taxation and requiring all cigarette packs be sold with graphic health warning labels (HWLs) that cover the top 50% of the front and back of the pack^4^. The Philippines’ Sin Tax Reform Act of 2012, for example, had a favorable impact on reducing demand and cigarette consumption at the population level^9^.

Despite these successes, there are gaps in the strength of policy coverage that may contribute to persistent high smoking rates in the country. For example, the current excise tax on cigarettes accounts for only 55% of the total retail price, which is well below the 70% recommended by the WHO^4^. Cigarettes continue to remain affordable for purchase as household incomes steadily rise in the country^10^. In addition, the HWL size has not increased since 2016, when the 50% HWLs were first implemented^11^. One study found that the current HWLs covering 50% of the front and back of the cigarette pack in the Philippines were less effective at encouraging individuals to quit smoking versus cigarette packs sold in plain packaging (i.e. no branding, single color presentation) with larger HWLs (e.g. 85% coverage)^12^. Further, there are currently no restrictions on the sale of flavored cigarettes in the Philippines. In 2012, roughly half (48%) of people who smoked manufactured cigarettes last purchased menthol cigarettes^1^, and evidence suggests that young adults in the Philippines perceive menthol cigarettes as more appealing and less harmful than non-flavored cigarettes^13^.

Global evidence suggests that raising the price of cigarettes through higher excise taxes^14^, increasing the size of health warnings^15-17^, implementing plain packaging^15,17^, and removing menthol cigarettes from the market^18^ are policy approaches that have the potential to reduce smoking. However, there is a gap in understanding how Filipinos who smoke would respond to these different policy scenarios, and data on local response is needed to support national policy development^19^. In the present study, we utilized a discrete choice experiment (DCE) to assess the relative importance of different cigarette price, flavor, and packaging scenarios on smoking-related decisions among adults who smoke in the Philippines. DCEs have been widely used in tobacco control research to elicit preferences of individuals in hypothetical scenarios and inform policy development^20^. DCE-based evidence from low- and middle-income countries are important to inform country-specific research gaps related to smoking cessation and tobacco control policies^20^. This includes countries with similar policy landscapes, like Vietnam, where a recent DCE study demonstrated the effect of plain packaging, larger HWLs, and higher excise taxes on increasing hypothetical quit intentions among Vietnamese people who smoke^21^. The present study aims to utilize a similar approach to estimate the relative importance of taxation, plain packaging, increased HWLs, and restricting menthol cigarette sales on smoking-related outcomes, behaviors, and perceptions among adults who smoke in the Philippines.

## METHODS

### Sample

Data were collected in November 2023 via an online survey. The Manila-based market research firm, GoodThinking, managed recruitment from an online panel maintained by Pureprofile. An invitation to complete the survey was sent to all Pureprofile panel participants who were aged ≥18 years, had complete demographic information, and had an IP address located in the Philippines. Participants were eligible if they smoked at least 100 cigarettes in their lifetime, smoked on at least one of the past 30 days, reported ever buying their own cigarettes, and were able to speak and read English, Filipino/Tagalog, or Cebuano. Quotas were set to recruit a sample of 900 adults who smoke stratified equally across the three main regions of the Philippine [Luzon (n=300), Visayas (n=300), Mindanao (n=300)]. Our sample size was calculated based on the simulations approach for DCEs^22^; we were adequately powered to detect differences in the main effects assuming a significance level of 0.05, power level of 80%, and medium effect sizes (0.2–0.4).

In total, 911 Filipino adults completed the survey. Participants took the survey in their language of choice: English, Filipino/Tagalog, or Cebuano. We removed 25 participants from the sample who completed the survey in under 4 minutes, or roughly one standard deviation less than the mean completion time of 10 minutes. This cut-point was determined based on survey length and prior experience of the research team in survey administration. It also aligns with best practices for removing ‘too fast’ responders to improve data quality and precision of the outcome estimates^23^. Based on our prior survey administration experience and best practices, our final analytic sample was 886 adults who currently smoke; about one-third resided in each of the three main regions of the Philippines (Luzon, Visayas, and Mindanao).

All participants provided digitally written consent to participate. The study protocol was approved by the Johns Hopkins Bloomberg School of Public Health Institutional Review Board (#00024497) and the Philippine Social Science Council Ethics Review Board (CF-23-24).

### Discrete choice experiment design and protocol

A DCE is designed so that individuals make hypothetical choices (e.g. purchase a product) based on a randomly assigned set of alternatives known as a ‘choice set’. Each alternative is defined by several levels or attributes. In a DCE approach, we assume that a respondent will choose the alternative that provides the highest individual benefit^20^. We then estimate the relative influence of each attribute (e.g. 20% increase in product price vs 10% increase) on the choices made (e.g. purchase a product) by looking across the choices made by all respondents.

We used a balanced, orthogonal fractional factorial design to construct 10 choice sets with three alternatives (power=80%, d-efficiency=90%). Power reflects the probability of finding a ‘true effect’ in the analysis given the design, while d-efficiency is a measure of how efficiently the design on a scale of 0–100% will be in estimating the outcomes compared to an ideal, hypothetical design.

Table 1 summarizes the choice sets and distribution across participants. Within each choice set, participants viewed three cigarette packs displayed at the same time. Packs varied on three attributes: 1) excise tax level – 60 Philippine pesos (PHP, reference), 70 PHP, 80 PHP, 90 PHP; 2) packaging design – branded ‘domestic’ pack (ref.), 50% graphic HWL coverage; plain ‘domestic’ pack, 50% graphic HWL coverage; branded ‘domestic’ pack, 85% graphic HWL coverage; plain ‘domestic’ pack, 85% graphic HWL coverage; branded ‘foreign’ pack, no HWL coverage; and 3) flavor – tobacco (ref.), menthol. Our sample size was large enough to allow us to randomize (using a 1:1 randomization ratio) each participant to view one choice set^24^. This helped to reduce participant burden^25^ while optimizing exposure to different pack attributes and capturing participants’ first instinct response^26^. We selected the levels of each attribute based on global best practices for product packaging, HWL coverage, and flavor regulations^14-18^, as well as the current tobacco control environment in the Philippines^4^.

**Table 1:** Choice sets by number block, cigarette pack attributes, and number of participants who viewed each choice set during a discrete choice experiment survey conducted among adults who smoke in the Philippines in November 2025 (N=886)

*Choice set*	*Pack^a^*	*Excise tax*	*Packaging*	*Flavor*	*Number of participants who viewed*
			*Branding | ‘Origin’^b^ | HWL size*		
1	1	60 PHP	plain | ‘domestic’ | 50% HWL	tobacco	122
	2	80 PHP	branded | ‘domestic’ | 85% HWL	menthol	
	3	70 PHP	branded | ‘domestic’ | 50% HWL	menthol	
2	1	60 PHP	branded | ‘foreign’ | no HWL	tobacco	104
	2	90 PHP	branded | ‘domestic’ | 85% HWL	menthol	
	3	70 PHP	plain | ‘domestic’ | 85% HWL	menthol	
3	1	80 PHP	branded | ‘foreign’ | no HWL	menthol	89
	2	90 PHP	branded | ‘domestic’ | 50% HWL	tobacco	
	3	60 PHP	plain | ‘domestic’ | 50% HWL	menthol	
4	1	90 PHP	plain | ‘domestic’ | 85% HWL	menthol	86
	2	80 PHP	branded | ‘domestic’ | 50% HWL	menthol	
	3	70 PHP	branded | ‘domestic’ | 85% HWL	tobacco	
5	1	60 PHP	branded | ‘domestic’ | 50% HWL	menthol	81
	2	70 PHP	plain | ‘domestic’ | 50% HWL	menthol	
	3	80 PHP	plain | ‘domestic’ | 85% HWL	tobacco	
6	1	80 PHP	branded | ‘domestic’ | 50% HWL	tobacco	82
	2	70 PHP	plain | ‘domestic’ | 85% HWL	tobacco	
	3	60 PHP	branded | ‘foreign’ | no HWL	menthol	
7	1	90 PHP	plain | ‘domestic’ | 50% HWL	tobacco	77
	2	80 PHP	branded | ‘domestic’ | 50% HWL	tobacco	
	3	60 PHP	plain | ‘domestic’ | 85% HWL	menthol	
8	1	90 PHP	branded | ‘domestic’ | 85% HWL	tobacco	86
	2	60 PHP	branded | ‘domestic’ | 50% HWL	tobacco	
	3	80 PHP	plain | ‘domestic’ | 50% HWL	menthol	
9	1	60 PHP	branded | ‘domestic’ | 85% HWL	tobacco	84
	2	90 PHP	branded | ‘domestic’ | 50% HWL	menthol	
	3	70 PHP	branded | ‘foreign’ | no HWL	tobacco	
10	1	70 PHP	branded | ‘domestic’ | 85% HWL	menthol	75
	2	90 PHP	branded | ‘foreign’ | no HWL	tobacco	
	3	60 PHP	plain | ‘domestic’ | 85% HWL	tobacco	

PHP: Philippines peso. HW: health warning label.

a Participants viewed all three packs at the same time in the numbered order listed.

b Two brands were created for the study and were designed to look similar to a ‘domestic’ and a ‘foreign’ cigarette pack currently sold in the Philippines.

For the excise tax level, we started by calculating the base price to which we would add the excise tax. The base price was estimated based on the participant’s response to one of two questions. If a participant knew how much they paid for their last cigarette purchase (reported in X number of cigarette sticks/packs (20)/pack (not 20)/carton), they were asked: ‘How much money did you pay for this purchase?’. If the participant did not know how much they paid for their last cigarette purchase, they were asked: ‘Think about the last time you or someone you know purchased a pack of cigarettes in the past 3 months. How much was the pack purchased?’. If a participant provided a last purchase price for a 20-cigarette pack in response to either question, this was the base price that was used in their DCE choice set. If a participant provided a last purchase price for a quantity other than 20 cigarettes, then the amount was standardized to a 20-cigarette pack price (e.g. 5 PHP for a one single stick=100 PHP for a pack of 20 cigarettes) and this was used as the base price in their DCE choice set. Finally, if a participant could not report an answer to either question, or if they provided a price for a 20-cigarette pack that was over 1000 PHP and considered out of the typical range, then the survey was programmed to use the average price for a pack of cigarettes (111 PHP) as the base price^27^. The excise tax levels were based on the current excise tax rate (60 PHP) or incremental increases to the current tax level (70, 80, and 90 PHP). The price of each pack shown in the choice set was calculated based on the base price plus assigned excise tax level: 60 PHP tax (base price only; current excise tax already included), 70 PHP tax (base price + 10 PHP), 80 PHP tax (base price + 20 PHP), and 90 PHP (base price + 30 PHP).

**Figure 1:**
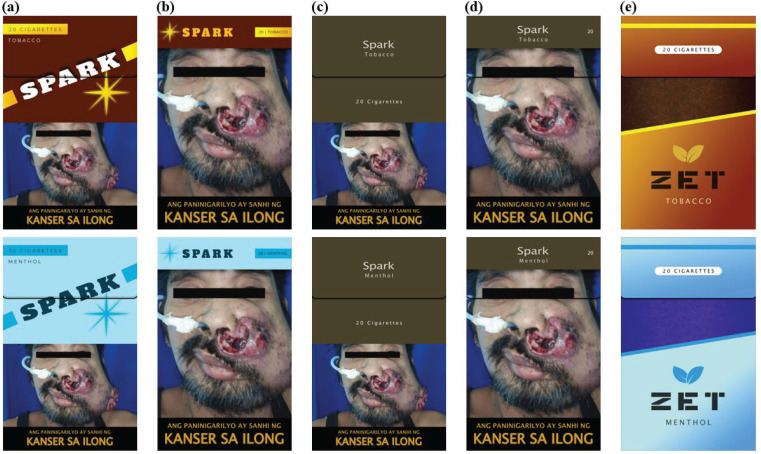
Mock cigarette pack images used in the discrete choice experiment survey conducted among adults who smoke in the Philippines in November 2025: a) branded ‘domestic’ pack with 50% health warning label (HWL); b) branded ‘domestic’ pack with 85% HWL; c) plain ‘domestic’ pack with 50% HWL; d) plain ‘domestic’ pack with 85% HWL; e) branded ‘foreign’ pack without an HWL. The top row contains the tobacco flavor packs, while the bottom row contains the menthol flavor packs

Packaging design levels were based on the current regulations (50% graphic HWL coverage, no plain packaging)^4^, and recommendations for increasing HWL coverage to 85% and implementing plain packaging^12^. For the packs with a HWL (either 50% or 85% coverage), we used the same graphic HWL of nose cancer that was currently in rotation in the Philippines on each pack (Figure 1)^28^. Flavor only had two levels (tobacco, menthol), which reflected the most popular flavored cigarette sold in the Philippines^1^.

We created two cigarette brands for the study stimuli to reduce any bias associated with an existing brand. The two created brands – Spark and Zet – were based on cigarette brands already sold in the Philippines. Spark was meant to represent ‘domestic’ cigarettes for sale in the country, while Zet was meant to represent a ‘foreign’ brand. For all Spark packs, we varied the excise tax added to the pack price, the branding and graphic HWL coverage, and flavor. For the Zet pack, we only varied the price and pack flavor; the pack was branded without an HWL to reflect packs that may be illicitly available for sale under any policy change condition.

### Outcome measures

Participants were shown their randomly assigned DCE choice set. Similar to other DCE studies^20,29^, we asked participants to consider which pack they would choose along with other behavior- or perception-related questions. In total, participants answered the following three questions in order for the cigarette packs in their choice set: 1) ‘If these were the only packs you could access, which one would you choose?’ with response options: Pack 1, Pack 2, Pack 3, and I would quit; 2) ‘Which one of these packs make you most think about quitting smoking?’ with response options: Pack 1, Pack 2, Pack 3, None of these packs; 3) ‘Which one of these packs do you think would be less harmful?’ with response options: Pack 1, Pack 2, Pack 3, None of these packs. The inclusion of ‘I would quit’ and ‘None of these packs’ followed best practices and provided respondents with the option to opt out of selecting any of the three packs presented^20^. For the first question, ‘I would quit’ was used as a response option instead of ‘None of these’ because the question was framed to force the participant to think of the three packs as the only packs available to access and smoke; not choosing or selecting any pack would mean the participant would hypothetically quit in the scenario. For the second and third questions, ‘None of these’ reflects the insufficiency of any pack presented in the choice set to make the participant think about quitting (a precursor to quitting^30^) or view a product as less harmful to use than the others in the choice set. Including the ‘I would quit’ or ‘None of these’ outcomes in the models mimics a marketplace where consumers always have the option to buy nothing and provides insight into the choices of individuals who might be most responsive to a tobacco control policy change.

### Analysis

Analyses were conducted using the *choice models (cm)* package in Stata version 16. Our outcomes were based on the three questions: 1) intention to choose a pack, 2) intention to quit smoking, and 3) least harmful. We used descriptive frequencies to characterize our study sample and outcomes. Given that participants made a single selection per outcome measure, this was a between-subjects design consisting of independent observations rather than repeated measures from the same individual. As such, we used the conditional logit choice model to estimate odds ratios (ORs). All models met the assumptions of independence of irrelevant alternatives using the Hausman specification test (p-values ranged from 0.1368 to 0.2216), and McFadden’s Pseudo R-square to assess goodness of model fit ranged from 0.20 to 0.22, indicating a very good fit of the model for the data. All models controlled for the following characteristics that are related to different patterns in smoking and could be related to the study outcomes: age (18–24, 25–34, ≥35 years), gender (man, woman, other/refuse), annual household income (HPH) (<60000, 60000–299999, ≥300000 PHP), and nicotine dependence (low, medium, high). Nicotine dependence was based on the two Heaviness of Smoking Index (HSI) questions – time to first cigarette and number of cigarettes smoked per day – which were used to calculate their nicotine dependence score as low, medium, or high^31^. For each outcome, we report the proportion of participants who selected a pack from the choice set and those who selected ‘I would quit’/‘None of these’. We also report the adjusted ORs (AORs) and 95% confidence interval (95% CI) for selecting the pack versus not selecting the pack by the independent effect of each attribute on the outcome: excise tax level (ref. 60 PHP), packaging (ref. branded ‘domestic’ pack, 50% coverage), and flavor (ref. tobacco). In all models, selecting ‘I would quit’ or ‘None of these’ was coded as not selecting a pack. All tests of association were two-tailed (p<0.05).

*Sensitivity and sub-group analyses*

We conducted a sensitivity analysis to assess the robustness of our findings against the imbalance in participant distribution across the ten choice sets (Table 1). We generated a restricted, balanced analytic sample by randomly down-sampling participants within each choice set block to equal the size of the smallest block (n=75) and re-estimated the conditional logit models. We also conducted sub-group analyses to see if there were differences in patterns of behavior based on region (Luzon, Visayas, Mindanao) and age (18–24, 25–34, ≥35 years). There were no major differences in the pattern of response by region or age group, and we report only the main effects across all participants.

## RESULTS

### Sample description

Table 2 presents descriptive statistics for participant demographics and smoking-related behavior. Approximately 25% of participants were aged 18–24 years, 41.5% were aged 25–34 years, and 33.6% were aged ≥35 years. Participants were relatively evenly represented across the three income categories, and the majority identified as male (57.2%). Most participants reported low (65.2%) or moderate (30.9%) levels of nicotine dependence.

**Table 2:** Demographic characteristics and smoking behaviors among participants in a cross-sectional discrete choice experiment survey conducted among adults who smoke in the Philippines, November 2025 (N=886)

*Characteristics*	*n (%)*
**Age** (years)	
18–24	220 (24.8)
25–34	368 (41.5)
≥35	298 (33.6)
**Gender**	
Man	507 (57.2)
Woman	369 (41.7)
Other	9 (1.0)
Refuse to answer	1 (0.1)
**Annual household income** (PHP)	
<60000	310 (35.0)
60000–299999	315 (35.6)
≥300000	252 (28.4)
**Nicotine dependence level^a^**	
Low	578 (65.2)
Moderate	274 (30.9)
High	32 (3.6)
**Price of last purchased cigarette pack** (PHP), mean (SD)	153 (268)
**Last cigarette purchase menthol flavored**	
Yes	662 (74.7)
No	223 (25.2)
**Tried to quit smoking in past 12 months**	
Yes	633 (71.4)
No	250 (28.2)
Refuse to answer	3 (0.3)

PHP: Philippine peso.

a Based on the Heaviness of Smoking Index calculated using reported time to first cigarette and number of cigarettes smoked per day.

On average, participants reported spending 153 PHP (SD=268), approximately US$2.70, on their last cigarette pack purchase. Around three-quarters of participants (74.7%) reported that the last pack that they purchased was menthol flavored, and most (71.4%) had tried quitting smoking in the past 12 months.

### Effect of price, packaging, and flavor on smoking-related outcomes

*Intention to choose a pack*

Table 3 presents findings from the DCE analyses. In response to the question, ‘If these were the only packs you could access, which one would you choose?’, 81.2% of participants selected a pack, and 18.8% selected ‘I would quit’. In the regression model, participants had 42%, 67%, and 76% lower odds of choosing a pack whose price included an excise tax of 70, 80, or 90 PHP versus the current 60 PHP excise tax rate, respectively (all p<0.001). With respect to packaging, participants had significantly higher odds of choosing the branded ‘foreign’ pack without a HWL versus the branded ‘domestic’ pack with 50% HWL coverage (AOR=4.02; 95% CI: 2.86–5.66). Flavor was not significantly associated with intention to choose a pack.

**Table 3:** Results from a discrete choice experiment survey conducted among adults who smoke in the Philippines in November 2025 of (a) intention to choose cigarette pack, (b) cigarette pack that most makes them think about smoking, and (c) cigarette pack they think is less harmful (N=886)

	*Intention to choose pack*		*Think about quitting smoking*		*Least harmful*	
	*% (n)*		*% (n)*		*% (n)*	
**Response distribution**						
Selected a pack from the choice set	81.2 (719)		87.4 (774)		71.4 (633)	
Selected ‘I would quit’^a^ or ‘None of these’^b^	18.8 (167)		12.6 (112)		28.6 (253)	
	***AOR (95% CI)***	***p***	***AOR (95% CI)***	***p***	***AOR (95% CI)***	***p***
**Excise tax level**						
60 PHP ®						
70 PHP	0.58 (0.45–0.74)	<0.001	0.86 (0.66–1.13)	0.279	0.69 (0.52–0.92)	0.011
80 PHP	0.33 (0.25–0.43)	<0.001	1.53 (1.18–1.99)	0.001	0.73 (0.56–0.96)	0.025
90 PHP	0.24 (0.18–0.33)	<0.001	1.94 (1.50–2.51)	<0.001	0.67 (0.50–0.89)	0.007
**Packaging**						
Branded ‘domestic’ pack, 50% HWL ®						
Plain ‘domestic’ pack, 50% graphic HWL	0.97 (0.73–1.27)	0.802	0.97 (0.69–1.36)	0.859	0.78 (0.57–1.06)	0.115
Branded ‘domestic’ pack, 85% HWL	0.79 (0.57–1.09)	0.156	3.40 (2.58–4.46)	<0.001	0.87 (0.64–1.19)	0.388
Plain ‘domestic’ pack, 85% HWL	0.85 (0.61–1.19)	0.352	3.30 (2.42–4.49)	<0.001	0.73 (0.51–1.04)	0.084
Branded ‘foreign’ pack, no HWL	4.02 (2.86–5.66)	<0.001	0.68 (0.47–0.99)	0.044	5.21 (3.70–7.32)	<0.001
**Flavor**						
Tobacco ®						
Menthol	0.96 (0.79–1.17)	0.714	0.84 (0.70–1.00)	0.053	1.06 (0.86–1.30)	0.591

AOR: adjusted odds ratio; models controlled for age, gender, income, and nicotine dependence level from the Heaviness of Smoking Index. ® Reference categories. PHP: Philippine peso. HWL: health warning label.

a Opt-out response for the question: ‘If these were the only packs you could access, which one would you choose?’.

b Opt-out response for the questions: ‘Which one of these packs make you most think about quitting smoking?’ and ‘Which one of these packs do you think would be less harmful?’.

*Think about quitting smoking*

When asked which of the three packs most makes them think about quitting smoking, 87.4% of participants selected a pack, and 12.6% selected ‘None of these’. In the regression model, participants had significantly higher odds of selecting the pack whose price included an 80 PHP (AOR=1.53; 95% CI: 1.18–1.99) and 90 PHP (AOR=1.94; 95% CI: 1.50–2.51) excise tax versus a 60 PHP excise tax. Participants also had significantly higher odds of selecting the branded (AOR=3.40; 95% CI: 2.58–4.46) and plain (AOR=3.30; 95% CI: 2.42–4.49) ‘domestic’ packs with 85% HWL coverage compared to the branded ‘domestic’ pack with 50% HWL coverage. Participants had significantly lower odds of selecting the branded ‘foreign’ pack without a HWL as the pack that most made them think about quitting smoking (AOR=0.68; 95% CI:0.47–0.99). Flavor was not associated with thinking about quitting.

*Least harmful*

When asked which of the three packs they thought would be least harmful, 71.4% of participants selected a pack, and 28.6% selected ‘None of these’. In the regression model, participants had lower odds of selecting the pack with a 70 PHP (AOR=0.69; 95% CI: 0.52–0.92), 80 PHP (AOR=0.73; 95% CI: 0.56–0.96), and 90 PHP (AOR=0.67; 95% CI: 0.50–0.89) excise tax added to the price versus the 60 PHP excise tax. Participants had significantly higher odds of selecting the branded ‘foreign’ pack without a HWL (AOR=5.21; 95% CI: 3.70–7.32) as least harmful compared to the branded ‘domestic’ pack with 50% HWL coverage. Flavor was not associated with perceived harm.

### Sensitivity analysis

To account for the imbalance in the distribution of choice sets by participants (Table 1), we re-ran the models with a restricted sample evenly balanced by choice sets. Sensitivity analysis estimates were similar in terms of magnitude and direction to the original main effects estimates reported in Table 3.

## DISCUSSION

Results from this study offer several important insights into potential pathways to strengthen tobacco control in the Philippines. One key finding from this study is that increasing the price of cigarettes by increasing the current excise tax from 60 PHP to 80 or 90 PHP could reduce the intention to purchase (i.e. choose) a pack and increase thoughts of hypothetical quitting and harm perceptions among Filipino adults who smoke. We also found that the presence of 50% HWL on a branded pack was effective at reducing intentions to choose/purchase a pack and increasing harm perceptions compared to a branded pack without a HWL. Further, people who smoke reported greater odds of thinking about quitting smoking when the HWL increased from 50% to 85%. Overall, our findings demonstrate the potential positive benefit of increasing the excise tax and the size of HWLs in terms of reducing possible cigarette consumption among people who smoke in the Philippines.

Our findings add to the existing literature that decreasing the affordability of cigarettes by increasing the cigarette price through taxation can help people who smoke in low- and middle-income countries quit^21^. In July 2019, the Philippines passed legislation which increased the excise tax on cigarettes to 45 PHP on 1 January 2020 and to 60 PHP by 2023; to match inflation, the tax rate was set to annually increase by 5% beginning 1 January 2024^32^. Research indicates that the Philippine’s 2019 cigarette taxation strategy has been cost-effective, and the country saved approximately US$367 million from the additional tax revenue and the reduced national healthcare costs associated with of smoking-related hospitalizations^33^. It is likely that increasing the excise tax to 80 or 90 PHP could provide further economic benefit at the population level, and, among people who smoke, increasing the excise tax by at least 20 PHP has the potential to reduce pack purchases, increase thoughts of quitting, and enhance perceived product harm.

Findings from this study also indicate an opportunity to advance packaging regulations and reduce the influence of illicit foreign packs without proper HWLs in the Philippines. Our data show that HWLs remain an important tool to warn about the dangers of combustible tobacco use among people who smoke in the Philippines. We found that participants viewed the branded foreign pack without a HWL as significantly less harmful than the branded domestic pack with 50% HWL coverage. Measures, such as product track and trace programs, can reduce illicit trade of foreign cigarette packs in the Philippines and hinder their potential to undermine tobacco control policies related to both packaging and price^34^.

Further, we found that increasing the HWL from 50% to 85% coverage – regardless of whether the pack is branded or a standard plain pack – could contribute to increasing intentions to quit among people who smoke. This aligns with global evidence that graphic health warnings that exceed 50% coverage increase intentions to quit smoking^17^. Our study results also complement a prior study conducted in the Philippines, which found that people across smoking statuses viewed plain packs with 85% HWL coverage as more effective at motivating quit attempts versus branded packs with 50% HWL coverage^12^. However, in our study, we found that branded and plain packs with an 85% HWL performed similarly at promoting thoughts of quitting compared to branded packs with 50% HWL coverage. One possible explanation is that our sample was comprised exclusively of Filipino adults who smoke, and plain packaging has been found to be more effective at reducing smoking initiation than increasing smoking cessation^34^. Collectively, our study and this prior research suggest that a policy combining an increase in HWL coverage to 85% with plain packaging would offer the greatest population-level benefit in the Philippines^12,34^.

We did not observe any differences in our study outcomes based on whether a pack was tobacco or menthol flavored. Instead, study participants were primarily driven by price and packaging when making their decisions. This is a surprising result given that nearly three-quarters of respondents reported that the last cigarette pack they purchased was menthol. It is possible that our manipulation of the pack text (tobacco versus menthol) or pack color (dark maroon/brown for tobacco versus blue for menthol) was too subtle for participants to notice compared to other pack features, like the warning labels. Additionally, the shades of blue used to represent menthol may not be the most common representation of menthol in the Filipino market, and the pack may not have been perceived to be a menthol pack or may not have been viewed as appealing as other menthol packs, including menthol capsule packs, on the market^13^. Nonetheless, there is a growing global evidence base on the effectiveness of menthol cigarette bans on reducing cigarette consumption and increasing smoking cessation^35^. Additional studies in the Philippines are needed to address the limitations of our design and generate country-specific evidence on how Filipinos who smoke would hypothetically respond to a national menthol ban.

### Limitations

This study is subject to several limitations. First, our outcomes are hypothetical and may not fully reflect the decisions that individuals make when a policy is implemented. Nevertheless, DCEs are a valuable approach for investigating behavioral responses to policies under consideration or not yet implemented. Second, we used a between-subjects, single-choice set DCE to reduce burden and over-exposure to choice attributes. An approach that exposed participants to several different choice sets could have provided more information and further refined our study estimates. Third, we only explored participants’ responses to cigarette packs and did not explore their responses to e-cigarettes or heated tobacco products, which are growing in popularity in the Philippines. Further, we showed participants two created or ‘fake’ cigarette brands instead of real cigarette brands to reduce the influence of any pre-existing experience or beliefs about a known cigarette brand on the study outcomes. Our brand design was based on a review of cigarette packs sold in the Philippines, purchased through the Tobacco Pack Surveillance System study^36^. However, it is possible that the created brands may not have appeared as realistic packs for purchase among participants, particularly the menthol variant. Third, the online survey was self-administered on each participant’s personal device. As such, we could not standardize the viewing experience of the study stimuli. It is possible that there was variation in image color or image resolution across participants and devices, which could have impacted responses. Additionally, although our study sample was equally distributed across the three regions of the Philippines and was similarly distributed in terms of age and wealth/income compared to the latest nationally representative data on Filipino adults who smoke^1^, this was not a nationally representative sample of Filipino adults who smoke and women, in particular, were over-represented compared to Global Adult Tobacco Survey (GATS) estimates^1^. Furthermore, individuals who are not online or have low digital literacy are under-represented in our sample. The smoking status of study participants was based on self-report and not biochemically verified, which can introduce misclassification bias to our estimates. In addition, our design may not include all potential confounders, and residual confounding may impact our study estimates. Although this study may inform policymakers and researchers in other countries facing similar smoking prevalence and tobacco control policy conditions, our results may not be fully generalizable.

## CONCLUSIONS

This study contributes to a growing body of local evidence to support cigarette tax and packaging policies in the Philippines. Our findings indicate that increasing the cigarette excise tax to at least 80 PHP and implementing 85% graphic HWLs on cigarette packs could potentially increase quit behavior among Filipino adults who smoke. Future studies can support this body of research by offering insights into how these policies may influence the potential appeal of cigarettes among people who do not smoke, particularly youth. Smoking prevalence in the Philippines remains one of the highest in the Western Pacific region^3^. Efforts to implement one or more tax and packaging-related policies could be a pathway to reduce smoking rates in the country and region.

## Data Availability

The data supporting this research are available from the authors on reasonable request.
